# Childhood Overnutrition among School Going Children in a Municipality: A Descriptive Cross-sectional Study

**DOI:** 10.31729/jnma.5367

**Published:** 2021-10-31

**Authors:** Alisha Thapa, Susmita Nepal, Garima Malla, Sushma Pokhrel

**Affiliations:** 1 Department of Public Health, Om Health Campus, Kathmandu, Nepal

**Keywords:** *children*, *obesity*, *overweight*, *prevalence*, *schools*

## Abstract

**Introduction:**

Childhood obesity, caused due to excessive fat accumulation, is one of the leading causes of preventable deaths associated with several non-communicable diseases. In Nepal, there is limited data available on the status of overweight and obesity among school children. The objective of this study was to find out the prevalence of childhood overnutrition among school going children in a municipality.

**Methods:**

A descriptive cross-sectional study was conducted from June 2019 to July 2019, in four schools of a municipality selected by simple random sampling. Ethical approval was obtained from Nepal Health Research Council (Registration number 380/2019). Data was collected using census sampling from children aged 5-18 years through self-administered questionnaires to the children's parents. The data was entered into the Statistical Package for Social Sciences for analysis. Point estimate at 95% confidence interval was calculated along with frequency and proportion for binary data.

**Results:**

Out of 379 school children, the prevalence of overnutrition was found to be 38 (10.03%) (95% Confidence Interval= 7.01-13.05). Prevalence of overnutrition was highest in children of age group 10-13 years 23 (60.5%), in females 27 (71.1%), those consuming junk food 4-6 times weekly 14 (36.8%) and those performing daily physical activity less than 60 minutes 24 (63.2%).

**Conclusions:**

The prevalence of childhood overnutrition is similar in comparison to other studies done in similar settings.

## INTRODUCTION

Overnutrition occurs when there is excessive fat accumulation which may present risks and impair the health of individuals.^1^ Childhood obesity is one of the leading causes of preventable deaths associated with several non-communicable diseases in children.^2^

Overweight and obesity are now on the rise in Nepal due to on-going urbanisation, economic transitions, shift in dietary patterns and lifestyle changes.^3^ However, adequate data on prevalence of overweight and obesity in school children is still lacking.^4^ The complications caused due to childhood obesity are severe which could continue to affect the health of an individual even in adulthood. Hence, this problem needs to be addressed at every possible step through effective interventions.^5^

The aim of this study is to find the prevalence of childhood overnutrition among school going children of a municipality.

## METHODS

A descriptive cross-sectional study was conducted in four schools of Budhanilkantha municipality, namely, Reliance Co. Ed. School, Sharada Academy, Shree Gram Sikshya Mandir Secondary School and Baluddhar Secondary School. Data collection period was one month from June 2019 to July 2019.

The ethical approval was obtained from Nepal Health Research Council (Reg. no. 380/2019) to conduct the study. Permission for data collection was received from the selected schools and written informed consent was obtained from parents/caretakers of study subjects.

School children present at the day of data collection and those within the age group 5 to 18 years were included in the study whereas all those children absent at the day of survey, those not included in the age criteria and children having chronic illnesses were excluded from the study.

A list of all the schools in Budhanilkantha municipality, Kathmandu was obtained from the municipality office and simple random sampling (lottery method) was applied to select the schools. The samples of 422 children were selected by using census method and the sample size was calculated by using the statistical formula,

n = Z^2^ × p × q / e^2^

  = (1.96)^2^ × 0.5 × 0.5 / (0.05)^2^

  = 0.9604 / 0.0025

  = 385

where,

n = sample size
Z = 1.96, at 95% confidence interval
p = prevalence taken as 50%
q = 1-p
e = margin of error, 5%

However, the total sample taken was 379.

The tools used during the survey were:

1. Weighing Machine: Weighing machine with the capacity of 100 kg and having the least count of 0.1 kg
2. Height measuring scale: A non-stretching measuring tape
3. Questionnaire: A well designed and pretested set of semi-structured questionnaire to collect information
4. WHO Growth Reference for children aged between 5-19 years

Semi-structured questionnaire was administered to the children's parents for data collection. Anthropometric measurements (height and weight) of children were recorded.

Anthropometric data was entered into WHO AnthroPlus to calculate BMI-for-age of each child. Overweight and obesity in children was determined based on cutoff values of the WHO Growth Reference for school aged children and adolescents (5-19 years). The data was then organized, coded and entered into Statistical Package for Social Sciences (SPSS) version 20 for analysis. Point estimate at 95% Confidence Interval and descriptive statistics using frequencies and percentages was calculated.

## RESULTS

Among 379 school children, the prevalence of overnutrition was found to be 38 (10.03%) (95% Confidence Interval= 7.01-13.05). Out of those 38 children, 31 (8.2%) were overweight and 7 (1.8%) were obese (Table 1).

**Table 1:** Distribution of respondents according to BMI and BMI-for-age (n=38).

Characteristics	n (%)
BMI-for-age	
Greater than +1 S.D. (Overweight)^*^	31 (8.2)
Greater than +2 S.D. (Obesity)^*^	7 (1.8)

* Overweight and obesity defined by WHO Growth Reference

Among 379 respondents, the mean age was 12.57±2.716 years. Age-wise, the prevalence of overweight/obesity was highest 23 (60.5%) in children of age group 10-13 years. Nearly three-fourth 27 (71.1%) and more than three-fourth 29 (76.3%) of the prevalence was seen in females and children with normal birth weight respectively (Table 2).

**Table 2:** Prevalence of overnutrition according to sociodemographic characteristics of respondents (n = 38).

Characteristics	Overnutrition n (%)
**Age (in years)**	
5-9	2 (5.3)
10-13	23 (60.5)
14-18	13 (34.2)
**Gender**	
Male	11 (28.9)
Female	27 (71.1)
**Perceived Birth weight**	
Low	4 (10.5)
Normal	29 (76.3)
High	5 (13.2)

The highest prevalence of overnutrition 16 (42.1%) was seen in those children who had 4 meals per day (Table 3). Children who consumed junk foods 4-6 times and those who consumed sugary beverages 2-4 times per week comprised more than one-third 14 (36.8%) and 15 (39.5%) respectively of the over nutrition prevalence (Table 3).

**Table 3:** Prevalence of overnutrition according to dietary habits of respondents (n = 38).

Characteristics	Overnutrition n (%)
**Number of meals per day**	
Less than 4 times	14 (36.8)
4 times	16 (42.1)
More than 4 times	8 (21.1)
**Junk food consumption per week**	
Never or rarely	0 (0.0)
1-3 times per week	13 (34.2)
4-6 times per week	14 (36.8)
Once a day or mor e	11 (28.9)
**Sugary beverages consumption per week**	
Never or rarely	2 (5.3)
Once per week	9 (23.7)
2-4 times per week	15 (39.5)
More than 4 times per week	12 (31.6)

Two-third 24 (63.2%) of the prevalence of overnutrition was seen in respondents who performed physical activities less than 60 minutes daily. Children who slept for 7-9 hours per day, accounted for nearly half 18 (47.4%) and those who had history of overnutrition in one of the parents accounted for two-fifth 15 (39.5%) of the prevalence of overnutrition (Table 4).

**Table 4:** Prevalence of overnutrition according to behavioural characteristics/genetic background of respondents (n=38).

Characteristics	Overnutrition n (%)
**Moderate to vigorous physical activities per day**	
Less than 60 minutes	24 (63.2)
60 minutes or more	14 (36.8)
**Sleeping hours**	
Less than 7 hours	16 (42.1)
7-9 hours	18 (47.4)
More than 9 hours	4 (10.5)
**Family history of overnutrition**	
Absent	13 (34.2)
Present in one of the parents	15 (39.5)
Present in both parents	10 (26.3)

## DISCUSSION

In this study, the prevalence of overweight and obesity among school children aged 5-18 years was found to be 10.03% out of which 8.2% were overweight and 1.8% were obese. This prevalence is higher than that found in a previous study conducted in Nepal among subjects aged 5-19 years in which out of 324 respondents, 10 (3.1%) were overweight and 2 (0.6%) were obese.^6^

A similar study done in private schools of Lalitpur district showed that altogether 25.9% school children were overweight and obese.^7^ This is quite higher than the results of present study.

Another study, done among school children of Biratnagar, revealed that 2.9% students were overweight and 1.8%, were obese.^4^ Although the prevalence of obesity is found to be similar, the prevalence of overweight is lower than that in the present study.

The prevalence of overweight and obesity among school children in Sylhet, Bangladesh was found to be 8.7% and 5.6% respectively.^8^ Similarly, a study in eastern state of India revealed that 8.9% students were overweight and 3.4% were obese.^9^

A cross-sectional pilot study conducted at a randomly selected private school in Bhaktapur showed that out of 83 students, 12% were either overweight or obese which is slightly higher than the prevalence found in the present study.^10^

Another cross-sectional study in Kaski district among adolescents showed the prevalence of overweight and obesity of 8.1% out of which 5.8% were overweight and 2.3% were obese.^11^

Similarly, the results of a study conducted in Iranian children and adolescents revealed that 9.7% were overweight and 11.9% were obese.^12^ This shows higher number of children were obese than overweight.

There were some limitations in the present study. As the study was cross-sectional, the prevalence of overnutrition might have been affected by seasonal variation. Also, this study explored the prevalence of childhood overweight and obesity among school children in Budhanilkantha municipality of Kathmandu district, so the findings cannot be generalized.

## CONCLUSIONS

The prevalence of childhood overnutrition is similar in comparison to other studies done in similar settings. As per the findings of this study, less than half of the school children are either overweight or obese which provide an assumptive severity of the problem of childhood overnutrition in Nepal. Other studies recommendes that parents discourage their children to have junk food and sugary beverages and encourage them to have nutritious foods such as fruits and vegetables. Schools are also recommended to prohibit the consumption of junk food in school.
